# Novel vegan ice cream made from red kidney bean milk with a probiotic: Technological and biofunctional characteristics

**DOI:** 10.1111/1750-3841.70087

**Published:** 2025-03-07

**Authors:** Emine Beyda Acar, Talha Karahan, Dora Mutlu, Osman Sağdıç, Hale İnci Öztürk

**Affiliations:** ^1^ Department of Food Engineering Konya Food and Agriculture University Konya Turkey; ^2^ Konya Sugar Industry and Trade Inc. Snack Products Factory Konya Turkey; ^3^ Department of Molecular Biology and Genetic Konya Food and Agriculture University Konya Turkey; ^4^ Department of Food Engineering Yıldız Technical University Istanbul Turkey

**Keywords:** functional food, *Phaseolus vulgaris*, phenolic content, plant milk, spore former probiotic

## Abstract

This study aimed to develop functional vegan ice creams (ICs) using red kidney bean milk (RKBM), with (RP) and without (R) probiotics (*Bacillus coagulans* ATCC 7050), and to compare their physicochemical, functional, and sensory properties with dairy counterparts. The IC samples had a dry matter content of 33.80%–35.54% and a protein content of 2.05%–2.20%. Although the brightness of the vegan samples was lower compared to dairy samples, the vegan samples were characterized by more reddish and yellowish hues. The vegan samples provided higher overrun values (36.00%–37.50%) compared to the dairy ones. Besides, the vegan samples exhibited higher antioxidant activity (5224–6148 µM Trolox equivalent antioxidant capacity/g) and total phenolic content (TPC) (72.98–184.75 mg gallic acid equivalent/100 g). The processes of ripening, fermentation, and freezing led to an increase in TPC values of all samples. The highest probiotic viability (7.52 log_10_CFU/g) was observed in the RP mix, but a decrease in probiotic cell count occurred after freezing. The vegan ICs received lower sensory scores than the dairy samples. Despite lower sensory scores, RKBM‐based ICs demonstrated suitable functional and technological properties, suggesting that RKBM has significant potential for use in functional vegan food products. Further studies are needed to improve sensory acceptability.

## 1 INTRODUCTION

In recent years, the sales of ice cream (IC) and frozen desserts have seen a slight increase, yet the category is evolving to feature more functional, non‐dairy, and “better‐for‐you” options (Mintel, 2019). The market for dairy‐free frozen desserts has notably expanded, with traditional IC brands now offering plant‐based alternatives. Consumers tend to choose non‐dairy ICs because of the perceived health advantages of plant‐based diets, as well as concerns about animal welfare and the environmental impact of dairy production (McCarthy et al., 2017).

These developments have played a role in the growth of the market for creative and health‐conscious products. The increasing demand for vegan and probiotic foods is among the significant developments. According to the Grand View Research (2022), the global vegan food market is anticipated to grow at a compound annual growth rate of 10.7% from 2023 to 2030. Additionally, probiotic products currently constitute 60%–70% of the functional food market (Küçükgöz & Trząskowska, 2022), and the Future Market Insights (2022) projects that the vegan probiotics segment will grow to USD 11.8 billion by 2032.

Probiotics are live bacterial cells that have benefits for the consumer when taken in sufficient amounts (Heenan et al., 2004). Probiotic *Bacillus* (*B*.) *coagulans* is highly resistant to food processing procedures, with its spores allowing it to survive harsher intestinal and gastric conditions compared to non‐spore‐forming probiotics (Cao et al., 2020). Studies have shown health benefits of *B. coagulans* consumption, such as improving immune system function and better gut microbiota control (Cao et al., 2022). Due to these properties, it has advantages over other probiotics and has significant potential to be incorporated in products such as IC.

Red kidney beans (RKBs), part of the Fabaceae family, are significant both economically and nutritionally as a global food crop. Known scientifically as *Phaseolus vulgaris*, they are highly valued in human diets for their rich complex carbohydrates (50%–60%), protein content (20%–25%), and being a great source of vitamins, minerals, polyunsaturated fatty acids, folate, and fiber (Punia et al., 2020). RKBs are rich in phenolics, functional proteins, and other active compounds with antioxidant, blood sugar, and fat‐lowering properties (Ganesan & Xu, 2017). Additionally, the α‐amylase inhibitor protein present in RKBs plays a crucial role in slowing starch digestion, thereby demonstrating potential for contributing to obesity prevention (Mojica et al., 2015). Although studies on plant‐based milks have focused on almond, coconut, soy, and rice milk varieties (Paul et al., 2020), RKB has been proposed as a new plant‐based milk source since 2023 (Aydar et al., 2023; Tang et al., 2023). In these studies, RKB milk (RKBM) was prepared using blanching, which can reduce nutritional properties, and there is a lack of literature regarding RKBM produced without blanching. This highlights the importance of extracting milk from raw beans followed by shorter duration pasteurization, as this approach preserves its nutritional properties and enhances its potential as an alternative to other plant‐based milks in various food applications. The increasing demand for vegan and probiotic foods reflects increasing awareness of how dietary choices impact overall well‐being. The current study aimed to meet these trends by developing vegan and probiotic IC formulations using RKBM as a plant‐based alternative and *B. coagulans* ATCC 7050 as the probiotic strain.

## 2 MATERIALS AND METHODS

### 2.1 Materials

The probiotic strain *B. coagulans* ATCC 7050 was obtained from Microbiologics™. The RKBs were purchased in dried form from a local market in Konya (Türkiye) in 2024. The skimmed cow's milk, xanthan gum, coconut oil, soy protein powder (70%), and skim milk powder used for IC production were procured from the brands İçim, Tito, Wefood, Vegrano, and Süt Çiftliği, respectively.

### 2.2 Production of RKBM

**FIGURE 1:**
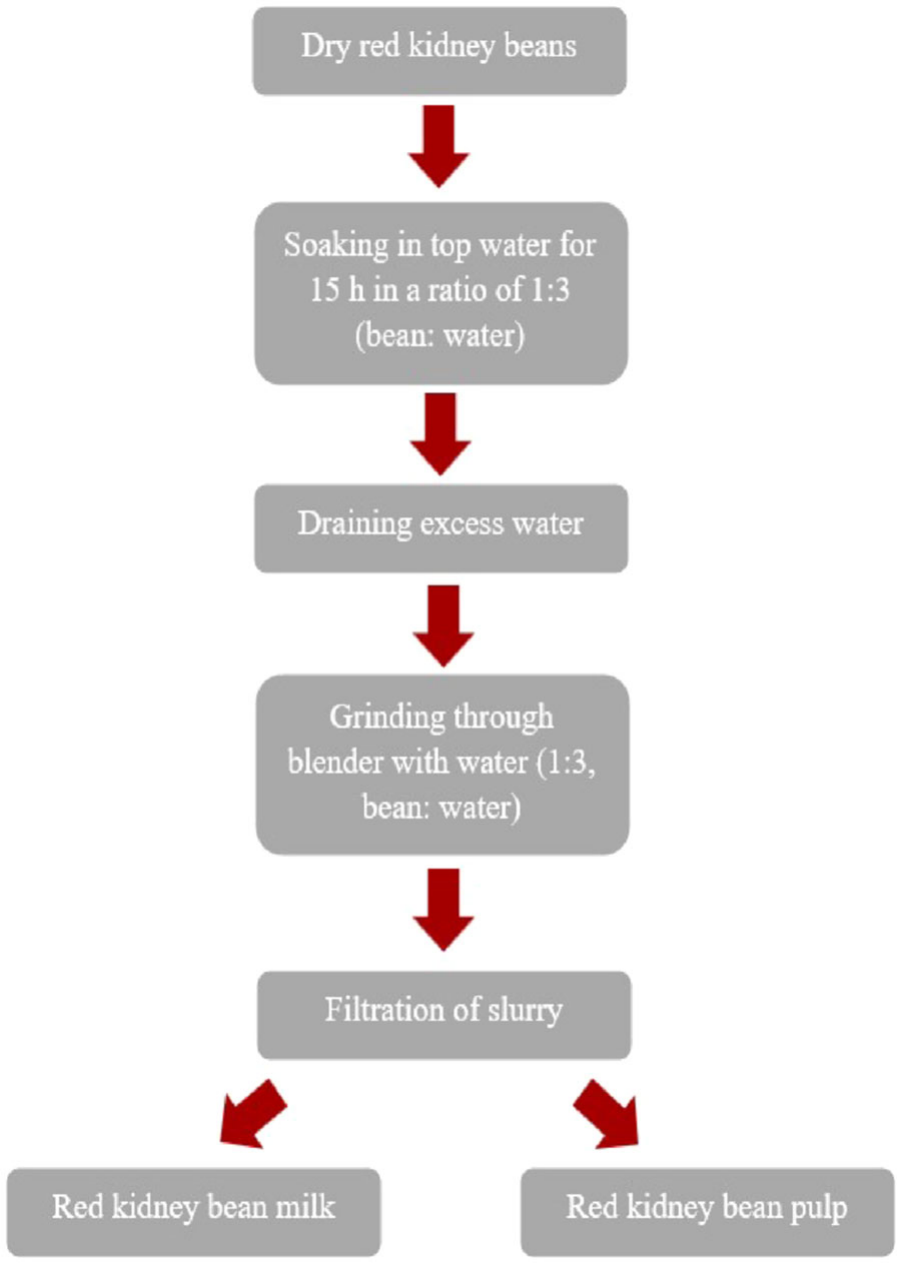
Production flow diagram for red kidney bean milk.

The production of RKBM was carried out by modifying the method recommended by Sethi et al. (2016) for soy milk. In this study, RKBM was produced without the application of a blanching treatment. The production flow diagram is presented in Figure 1. The resulting milk was pasteurized at 83°C for 5 min (Meghrabi & Yamani, 2023) and then stored at 4°C until IC production.

### 2.3 Analyses in RKBM

#### 2.3.1 Physicochemical properties

The pH of RKBM was recorded using a calibrated pH meter (315i/SET, WTW). For titratable acidity, 10 mL of milk sample was taken and titrated with 0.1 N NaOH until the pH reached 8.2. The titratable acidity was expressed as percentage of citric acid (Meghrabi & Yamani, 2023). Total soluble solids content was measured by a manual refractometer (ATC, BX50). Color evaluation of the RKBM was conducted using a colorimeter (Serlab SL‐400).

#### 2.3.2 Total phenolic content and antioxidant activity

To prepare the extracts for total phenolic content (TPC) and antioxidant activity analyses, a modified version of the method suggested by Aydar et al. (2023) was employed. To that end, 5 mL of milk sample was mixed with 25 mL of 80% methanol solution and incubated in a shaking incubator at room temperature for 2 h. Following the incubation, the samples were centrifuged at 3000 rpm for 15 min, and the supernatant was filtered through a membrane with a 0.45 µM pore size to obtain the extracts. The antioxidant activity of RKBM was evaluated using the DPPH method (Demirci et al., 2017). A standard Trolox curve was obtained by preparing Trolox solutions at different concentrations (0.1–10.0 mM). According to the Trolox curve, antioxidant activities were expressed in µM Trolox equivalent antioxidant capacity (TEAC) per mL of milk sample. The TPC in the extracts was assessed using the Folin–Ciocalteu procedure as described by Öztürk et al. (2018). The results, calculated with reference to the gallic acid curve (0.05–1.00 mg/mL), were presented as milligram gallic acid equivalents (GAEs) per 100 mL of milk samples.

### 2.4 Preparation of probiotic culture

*B. coagulans* ATCC 7050 was activated in MRS broth (Merck) at 37°C overnight under aerobic conditions to achieve a concentration of 10^8–9^ CFU/mL. After incubation, the activated culture was centrifuged at 4000 rpm for 15 min at 4°C. The resulting pellets were washed twice with sterile distilled water and then resuspended in sterile distilled water to achieve a final concentration of 0.02 g/mL in the mix.

### 2.5 IC manufacturing and experimental design

Various formulations were developed to standardize the IC mixes at a specific dry matter ratio, as shown in Table 1. Four IC samples were produced: dairy without *B. coagulans* (C), dairy with *B. coagulans* (CP), RKBM without *B. coagulans* (R), and RKBM with *B. coagulans* (RP). Both RKBM and skimmed cow's milk were used, with coconut oil as the fat source (8% fat content). Xanthan gum was added as an emulsifier, and sahlep as a stabilizer. Skim milk powder was used for dairy ICs, and soy protein for vegan ones. The ingredients were mixed, homogenized by a blender (Braun, MultiQuick 5) for 2 min, pasteurized at 85°C for 1 min, and cooled to 37°C. A probiotic culture (8.94 log_10_CFU/mL) at a concentration of 0.02 g/mL was added to the CP and RP mixes, which were incubated at 37°C until pH 5.5, whereas C and R groups aged overnight at 4°C. After fermentation, all mixes were frozen using an IC machine (Maraşmatik AG 30, Gürdal Machine), packaged, hardened and stored at −20°C.

**TABLE 1:** Formulated compositions of ice cream mixes.

Constituents (%)	Ice cream samples^a^			
	C	CP	R	RP
Sugar	16.00	16.00	16.00	16.00
Stabilizer	0.40	0.40	0.40	0.40
Emulsifier	0.20	0.20	0.20	0.20
Coconut oil	8.00	8.00	8.00	8.00
Soy protein	–	–	5.40	5.40
Red kidney bean milk	–	–	70.00	68.00
Skimmed milk powder	4.75	4.75	–	–
Skimmed milk	70.65	68.65	–	–
Probiotic strain	–	2.00	–	2.00

^a^ C: traditional dairy ice cream made from cow milk; CP: dairy ice cream supplemented with probiotic *B. coagulans*; R: vegan ice cream made from red kidney bean milk; RP: vegan ice cream supplemented with probiotic *B. coagulans*.

### 2.6 Analyses in IC samples

Physicochemical, microbiological, and sensory analyses were applied on hardened IC samples on Day 2. In addition, analyses for probiotic viability, antioxidant activity, and TPC were conducted after fermentation, ripening, and freezing processes.

#### 2.6.1 pH and titratable acidity

The pH analysis was conducted using a pH meter (315i/SET, WTW, Weilheim, Germany) according to the AOAC (2000) method (981.12). Titratable acidity was also determined following the AOAC (2000) method (947.05). The protein and dry matter contents of the IC samples were determined according to AOAC (2000) methods 930.33 and 941.08, respectively. Titratable acidity was expressed as a percentage of citric acid for vegan ICs and as a percentage of lactic acid for dairy ICs.

#### 2.6.2 CIELAB color measurements

Color analysis of the IC samples was performed using a colorimeter (Serlab SL‐400) to measure the three‐dimensional color space based on the CIELAB system.

#### 2.6.3 Dry matter and protein contents

The protein and dry matter contents of the IC samples were determined according to AOAC (2000) methods 930.33 and 941.08, respectively. All results were expressed as percentages.

#### 2.6.4 Overrun

Overrun was analyzed using the weight method, which involves measuring the mass of a fixed volume of the IC mix and comparing it to the mass of the same volume of the IC (Warren & Hartel, 2018). The results were presented as percentages.

#### 2.6.5 Probiotic enumeration

Viable bacterial counts were determined for *B. coagulans* ATCC 7050 using the spread plate method. MRS agar (Merck) was used for plating, and after inoculation, the plates were placed in an incubator at 37°C for 24–48 h under aerobic conditions (Lavrentev et al., 2021). Colony counts were enumerated following the incubation, and results were expressed as log_10_CFU/g.

#### 2.6.6 TPC and antioxidant activity

For the analysis of TPC and antioxidant activity analyses, extracts were first prepared from the IC samples. A modified version of the method suggested by Öztürk et al. (2018) was used for this purpose. To prepare the extracts, 5 g of the IC sample was combined with 25 mL of 80% methanol and then vortexed. The prepared mixture was kept at 25°C for 2 h. After centrifuging the samples at 3000 rpm for 15 min, the supernatant was collected and filtered using a 0.45 µM membrane filter to obtain the extracts. For the TPC analysis, the method proposed by Öztürk et al. (2018) for ICs was applied. The results were expressed as mg GAE/100 g, based on a calibration curve prepared with gallic acid. In the antioxidant activity analysis, the method of DPPH radical scavenging activity (Demirci et al., 2017) was applied, and the results were expressed as µM TEAC/g of IC sample.

#### 2.6.7 Sensory evaluation

A nine‐point hedonic scale, ranging from 1 (extreme dislike) to 9 (extreme like), was used for the sensory evaluation of the IC samples (Atallah et al., 2022). The sensory evaluation was conducted with 25 semi‐trained panelists (12 male and 13 female), aged between 19 and 50 years, consisting of students and academic staff from the campus. The panelists participated in prior training sessions, during which specific sensory attributes and evaluation criteria were defined and thoroughly discussed to ensure consistent understanding within the group. The IC samples were assessed by the panelists based on several attributes, including taste, odor, color, smoothness, lubricity, melt‐in‐mouth sensation, appearance, and overall acceptability. The IC samples (20 g) were transferred from their original containers to the plastic sensory cups at least 5 h prior to the evaluation, after which the cups were sealed with lids and stored at −15°C until serving. For the sensory evaluation, IC samples were served at −10°C with a transparent plastic spoon.

The study was reviewed and approved by the Yıldız Technical University IRB, and informed consent was gathered from all participants before they took part in the study.

### 2.7 Statistical analysis

All production experiments were conducted in duplicate, with each analysis repeated three times. The results are presented as mean values along with standard deviations. The data were analyzed using analysis of variance through Minitab software (version 17). The Tukey's multiple range test was applied to detect significant differences between samples and processing conditions, with a confidence level of 95%. Moreover, the probiotic cell counts between vegan and dairy samples were compared using an independent two‐sample *t*‐test.

## 3 RESULTS AND DISCUSSION

### 3.1 Physicochemical characteristics, TPC, and antioxidant activity of RKBM

The physicochemical properties of RKBM are presented in Table 2. The total soluble solids of RKBM, prepared with a 1:3 (w:v) water addition, were determined to be 7.23%. In the study of Aydar et al. (2023), the total soluble solids for RKBM from different varieties were reported to be between 3% and 4%. The differences between these studies could be attributed to the amount of water used during milk preparation and the variety of RKBs. Indeed, Tang et al. (2023) have reported that the variety of RKBs significantly impacts the soluble solid content. The pH of RKBM was lower than that of regular dairy milk, measuring 6.50, whereas the titratable acidity was identified as 0.14% in terms of citric acid. The pH of RKBM was slightly lower than that of soy milk (6.80), oat milk (7.16), and rice milk (7.47), based on the findings of Mäkinen et al. (2015).

**TABLE 2:** Physicochemical properties, total phenolic content, and antioxidant activity of red kidney bean milk.

Parameters		Red kidney bean milk
Total soluble solids (%)		7.23 ± 0.25
pH		6.50 ± 0.01
Titratable acidity (% citric acid)		0.14 ± 0.01
Color characteristics	L*	66.60 ± 1.44
	a*	5.10 ± 0.10
	b*	11.90 ± 0.14
Total phenolic content (mg GAE/100 mL)		57.64 ± 3.80
Antioxidant activity (µM TEAC/mL)		6882.62 ± 191.12

*Note*: Data are expressed as mean ± standard deviation (*n* = 2).

According to the color measurements, the L* value of RKBM was 66.50 with moderate brightness. Moreover, the a* value of 5.0, indicating slight redness, was determined in the RKBM. This color value can be attributed to the presence of natural red pigments such as anthocyanins, including pelargonidin 3‐glucoside, cyanidin 3,5‐diglucoside, cyanidin 3‐glucoside, and delphinidin 3‐glucoside, found in the beans (Choung et al., 2003). For almond, rice, coconut, soy, and oat milks, the a* values were reported as 1.04, −0.98, −1.04, −0.55, and −0.88, respectively (Daszkiewicz et al., 2024). Accordingly, the slight red coloration observed in this study is a characteristic feature that differentiates RKBM from other plant‐based milks. On the other hand, the b* value of 11.90 in RKBM, which indicates yellowness, was similar to the b* values observed in other plant‐based milks (Daszkiewicz et al., 2024). This yellowness value can be associated with the presence of carotenoids and yellow‐colored flavonoids like quercetin, luteolin, and kaempferol in RKBM (Yang et al., 2018).

According to Table 2, the TPC of RKBM was determined to be 57.64 mg GAE/100 mL. Unsurprisingly, this value was higher than the 14.14–21.64 mg GAE/100 mL range reported by Aydar et al. (2023) for RKBM extracted with a 1:6 water ratio. RKB has been reported to contain phenolic acids and flavonoids (Zhu et al., 2024). Among the phenolic acids, hydroxycinnamic and ferulic were dominant, whereas the most abundant flavonoids included flavanols (catechin), isoflavones (isogenistein 7‐glucoside), and anthocyanins (petunidin, pelargonidin, and cyanidin derivatives). Phenolic compounds have been shown to exhibit antioxidant activity by scavenging free radicals and preventing oxidative reactions through electron donation, known as reducing power (Mathew et al., 2015). The obtained RKBM exhibited DPPH radical scavenging activity with a value of 6882.62 µM TEAC/mL (Table 2). García‐Lafuente et al. (2014) have reported that catechins in RKBs significantly contribute to antioxidant activity. Besides, Mathew et al. (2015) have proposed that the multiple hydroxyl groups in catechins offer high free radical scavenging activity against DPPH radicals. The high TPC and potent antioxidant activity of RKBM suggest its potential as a valuable functional ingredient in the food industry.

### 3.2 Evaluation of the physicochemical characteristics of IC

The pH values of the mixes, fermented and ripened mixes, as well as the IC samples, are presented in Table 3. After the mixing process, the pH values of the mix were recorded between 6.33 and 6.45. As expected, a decrease in pH was observed in fermented (CP and RP) mixes (*p* ≤ 0.05). The pH decrease in the fermented mixes could be attributed to the production of organic acids and fatty acids by the *B. coagulans*. Regarding this, it has been reported that the conversion of lipids, sugars, and other compounds into fatty and organic acids occurs through the action of lipase and amylase enzymes produced by *B. coagulans* during fermentation (Pan et al., 2023).

**TABLE 3:** pH and titratable acidity changes in mix and ice cream samples.

Samples^*^	First mix	Fermented or ripened mix^**^	Ice cream
pH			
C	6.33 ± 0.01^C, c^	6.39 ± 0.01^B, b^	6.47 ± 0.00^B, a^
CP	6.30 ± 0.00^D, a^	5.75 ± 0.01^C, c^	5.79 ± 0.01^C, b^
R	6.45 ± 0.01^A, c^	6.50 ± 0.01^A, b^	6.53 ± 0.01^A, a^
RP	6.40 ± 0.01^B, a^	5.63 ± 0.01^D, c^	5.65 ± 0.00^D, b^
Titratable acidity^***^			
C	0.26 ± 0.01^A, b^	0.30 ± 0.02^B, a^	0.32 ± 0.01^C, a^
CP	0.25 ± 0.04^A, b^	0.52 ± 0.02^A, a^	0.52 ± 0.02^A, a^
R	0.21 ± 0.02^A, a^	0.20 ± 0.04^C, a^	0.25 ± 0.01^D, a^
RP	0.25 ± 0.02^A, b^	0.50 ± 0.00^A, a^	0.47 ± 0.02^B, a^

*Note*: Data are expressed as mean ± standard deviation (*n* = 2).

* C: with dairy milk and without *B. coagulans*; CP: with dairy milk and with *B. coagulans*; R: with red kidney bean milk and without *B. coagulans*; RP: with red kidney bean milk and with *B. coagulans*.

** The CP and RP mixes were fermented, whereas the C and R mixes were ripened.

*** Titratable acidity was calculated as a percentage of lactic acid for dairy samples and as a percentage of citric acid for vegan samples.

Different uppercase letters in the same column signify significant differences across samples, whereas different lowercase letters in the same row denote differences across treatments (*p* ≤ 0.05).

Previous studies found that *B. coagulans* resulted in a pH‐lowering effect by producing acid in plant‐based milks (Christian et al., 2021) and dairy products (Ma et al., 2021). Similarly, the higher titratable acidity in fermented CP and RP mixes (Table 3) compared to ripened mixes confirms the pH decline due to increased acidity. The greatest reduction in pH after fermentation was observed in mixes containing RKBM (*p* ≤ 0.05). This can be attributed to the fact that RKBM provided a more favorable environment for the growth of *B. coagulans*, leading to greater acid production. The highest viable count of *B. coagulans* was also determined in the fermented mixes containing RKBM. According to Table 3, the lowest pH was observed in both probiotic IC samples (pH 5.65–5.79) (*p* ≤ 0.05). Among IC samples, the highest pH value was observed in sample R (6.53), whereas the lowest pH value was determined in sample RP in IC (5.65) (*p* ≤ 0.05).

Leahu et al. (2022) underscored the strong correlation between titratable acidity and pH levels, noting that these factors play a crucial role in determining both product quality and consumer acceptance of plant‐based ICs. As expected, the titratable acidity values of the fermented mixes were higher than that of the ripened mixes (*p* ≤ 0.05). It was 0.50% citric acid for RP and 0.52% lactic acid for CP (Table 3). The effect of the freezing process on the titratable acidity of all ICs was found to be not statistically different (*p* > 0.05). The highest titratable acidity was also observed in the CP and RP ICs.

The dry matter content of IC samples was in the range of 33.80%–35.54% (Table 4). No significant difference was determined among the samples in terms of dry matter content due to the standardization (*p* > 0.05). The obtained dry matter values are consistent with the values reported in the literature for both plant‐based (31.35%–38.53%) and dairy (33.34%–38.59%) ICs (Akalın et al., 2024; Carvalho et al., 2022). The protein content of the IC samples ranged from 2.05% to 2.20%, with no statistically significant difference observed between the samples (*p* > 0.05) (Table 4). Protein content has been reported as 3.9%–4.6% in ICs made with yam milk (Batista et al., 2019), 3.35%–3.48% in those made with cow's milk (Carvalho et al., 2022), 1.09%–2.66% in hemp and almond milk ICs (Leahu et al., 2022), and 2.88% in sesame milk ICs (Ghaderi et al., 2021). The protein values obtained in this study are comparable to the protein content reported in previous studies for several ICs.

**TABLE 4:** Physicochemical characteristics of ice cream samples.

Samples^*^	Color characteristics			Overrun (%)	Dry matter (%)	Protein (%)
	*L* ^*^	*a* ^*^	*b* ^*^			
C	91.43 ± 1.04^A^	−2.48 ± 0.35^C^	10.48 ± 0.54^B^	18.50 ± 3.50^B^	35.54 ± 0.23^A^	2.10 ± 0.00^A^
CP	90.73 ± 0.62^A^	−1.58 ± 0.18^B^	10.00 ± 0.68^B^	22.50 ± 2.50^B^	33.83 ± 0.05^B^	2.05 ± 0.05^A^
R	74.35 ± 1.17^C^	3.60 ± 0.07^A^	15.23 ± 0.08^A^	37.50 ± 7.50^A^	34.92 ± 1.43^AB^	2.05 ± 0.05^A^
RP	79.70 ± 0.56^B^	3.33 ± 0.26^A^	15.45 ± 0.22^A^	36.00 ± 1.00^A^	33.80 ± 0.01^B^	2.20 ± 0.10^A^

*Note*: Data are expressed as mean ± standard deviation (*n* = 2).

* C: with dairy milk and without *B. coagulans*; CP: with dairy milk and with *B. coagulans*; R: with red kidney bean milk and without *B. coagulans*; RP: with red kidney bean milk and with *B. coagulans*.

Different uppercase letters in the same column signify significant differences across samples (*p* ≤ 0.05).

Color is a key attribute in IC products, as it significantly affects consumer perception and acceptance (Gorman et al., 2023). Color attributes of IC samples are demonstrated in Table 4. Dairy ICs were brighter (90.73%–91.43) (*p* ≤ 0.05), whereas the brightness decreased in ICs containing RKBM (74.35–79.70). It has generally been reported that ICs made with plant‐based milk have lower brightness (Akalın et al., 2024). Unlike ICs made with hazelnut, lupin, and almond milk, which had a* values ranging from −0.62 to −6.84 (Akalın et al., 2024), the vegan ICs made with RKBM in this study had a* values of 3.33–3.60, indicating a slight reddish hue. In dairy IC samples, the b* value was between 10.00 and 10.48, whereas this value was between 15.23 and 15.45 in vegan IC samples. Thus, it can be concluded that the yellow coloration was more pronounced in IC made with RKBM than in dairy IC.

Overrun refers to the percentage increase in IC volume due to the incorporation of air during the mixing motion applied in freezing process. In this stage, air bubbles become enveloped by fat globules within the emulsion system (Muhardina et al., 2019). Overrun is a key physical attribute of IC, influencing its texture by contributing to its softness through the retention of air. The overrun values of dairy ICs were determined between 18.50% and 22.50%, whereas vegan ICs showed an overrun of 36.00%–37.50%. The overrun values of ICs made from soy and sesame milk were found as 20%–30% (Ghaderi et al., 2021). In a study using different emulsifiers, the overrun values of vegan IC produced with rice milk were determined between 10.91% and 33.96% (Mygdalia et al., 2023). Compared to the findings in the literature, the overrun values obtained in this study were consistent with those from the previous research and, in some cases, even slightly higher. The high overrun values observed in vegan samples highlight the potential of RKBM as a valuable ingredient in the food industry for creating plant‐based ICs with enhanced air retention. This enhanced air retention may not only contribute to improving texture but also seems to align with industrial objectives for cost efficiency by potentially increasing product volume or yield.

The effect of probiotic addition on overrun was insignificant (*p* > 0.05). However, the use of plant‐based milk significantly influenced overrun, with RKBM‐based IC samples exhibiting higher air retention capacity than dairy ICs (*p* ≤ 0.05). To clearly highlight the impact of milk type, both vegan and dairy ICs were prepared using identical fat, emulsifier, and stabilizer contents. Air cell formation and stability in IC are known to depend on the fat, protein, emulsifier, and stabilizer content (Kasapoglu et al., 2023). Consistent with this study, Pontonio et al. (2022) reported higher overrun in plant‐based ICs, attributed to the emulsifying and foaming properties of legume proteins, which enhance air incorporation and stability. Additionally, fiber and polysaccharides in plant‐based milks, particularly legumes, contribute to increased mix viscosity, facilitating efficient air incorporation and stabilizing smaller air cells (Samakradhamrongthai et al., 2021). The higher overrun observed in RKBM‐based ICs can be attributed to the dietary fiber content of RKBs, which consists of 29.32%–46.77% fiber, primarily composed of mannose, galactose, galacturonic acid, and arabinose (Kan et al., 2017). Conversely, some studies suggest that increased viscosity could reduce overrun by hindering air incorporation during the IC‐making process (Sivasankari et al., 2019). These conflicting views may result from variations in IC production methods, such as differences in ripening times, and the specific characteristics of the freezing equipment used. Therefore, further studies are necessary to clarify these contradictory perspectives, considering all relevant production factors.

### 3.3 Antioxidant activity and TPC of IC samples in relation to production parameters

**FIGURE 2:**
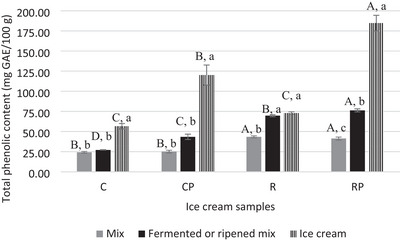
Total phenolic content (TPC) of ice cream samples and its variation according to ice cream production parameters. Data are expressed as mean ± standard deviation (*n* = 2). C: with dairy milk and without *Bacillus coagulans*; CP: with dairy milk and with *B. coagulans*; R: with red kidney bean milk and without *B. coagulans*; RP: with red kidney bean milk and with *B. coagulans*. The CP and RP mixes were fermented, whereas the C and R mixes were ripened. Different uppercase letters signify significant differences across samples, whereas different lowercase letters denote differences across treatments (*p* ≤ 0.05).

The present study evaluated the TPC and antioxidant properties at three stages of IC production: after mixing, after ripening or fermentation, and after freezing, as illustrated in Figure 2. Vegan ICs (41.21–43.40 mg GAE/100 g) exhibited significantly higher TPC compared to dairy ICs (24.22–25.00 mg GAE/100 g) (*p* ≤ 0.05). The phenolic content in dairy milk, consisting of flavonols, flavanols, phenolic acids, and flavones, has been reported to vary with the feed composition of dairy animals (Bennato et al., 2023). Predominant phenolic compounds include caffeic acid and rosmarinic acid among phenolic acids, epigallocatechin among flavanols, quercetin and kaempferol among flavonols, and luteolin and naringenin among flavones. Similarly, coconut oil is known to contain phenolics such as catechin, *p*‐coumaric acid, caffeic acid, syringic acid, and ferulic acid (Mulyadi et al., 2018), which may contribute to the TPC observed in dairy mixes. In contrast, RKBs are exceptionally rich in phenolic compounds, including phenolic acids, flavonoids, isoflavones, and anthocyanins (Zhu et al., 2024), accounting for the significantly higher TPC levels observed in IC samples made with RKBM compared to dairy‐based ICs.

After ripening of the normal dairy mix and fermentation of the probiotic dairy mix, TPC values were 27.12 and 43.40 mg GAE/100 g, respectively. Although TPC in dairy mixes slightly increased after ripening and fermentation, these changes were not statistically significant (*p* > 0.05). In contrast, RKBM‐based vegan mixes showed a significant increase in TPC after both ripening and fermentation (*p* ≤ 0.05), with fermented mixes having significantly higher TPC than ripened mixes (*p* ≤ 0.05). The observed increase in TPC content during the ripening period may be attributed to the activity of enzymes present in RKBM, which could break down plant cells, releasing more phenolic compounds from the cells or chemically bound phenolic components. Enzymes like β‐galactosidases and cellulases, previously identified in RKBs (Taha, 2024), play a crucial role in releasing phenolic compounds. β‐galactosidases facilitate the release of free phenolic compounds by separating glucose from phenolic compounds (Mulyadi et al., 2018). Additionally, cellulases can break down cell membranes, leading to the release of cellular contents such as phenolic compounds (De Faveri et al., 2008). The increased TPC content found in the mixes fermented with probiotic *B. coagulans* ATCC 7050 could be linked to microbial metabolic activity. During fermentation, *B. coagulans* can produce enzymes, such as carboxymethylcellulase (Odeniyi et al., 2009), polygalacturonase (Odeniyi et al., 2009), cellulase (Orji et al., 2016), and pectinase (Raj Kashyap et al., 2003), which break down plant cell walls, leading to the formation of phenolics. Furthermore, this microbial species is known to produce β‐galactosidase, which separates glucose from phenolic glycosides, resulting in the release of free phenolic compounds (Orji et al., 2016). As the fermentation temperature of the mixes was near the optimal range for these enzymes, the higher TPC values in fermented mixes may have resulted from bacterial enzyme activity.

After freezing, the TPC of dairy IC samples increased to 56.54–120.10 mg GAE/100 g, whereas vegan ICs ranged from 72.98 to 184.75 mg GAE/100 g. Comparatively, TPC values reported for plant‐based ICs include 9.30 mg GAE/100 g for coconut milk ICs (Perera & Perera, 2021), 51–54 mg GAE/100 g for yam‐based ICs (Batista et al., 2019), and 30.61–42.30 mg GAE/100 g for almond and hazelnut milk ICs (Akalın et al., 2024). In this study, RKBM‐based ICs exhibited generally higher TPC levels than those reported in the literature. The highest TPC was observed in probiotic vegan ICs, followed by probiotic dairy ICs (*p* ≤ 0.05), indicating a positive effect of probiotics on phenolic content. Freezing also increased TPC in all samples, likely due to ice crystal formation causing cell rupture and releasing bound phenolics into the matrix (Sun et al., 2023). Overall, both fermentation and freezing contributed to enhancing the TPC of ICs.

**FIGURE 3:**
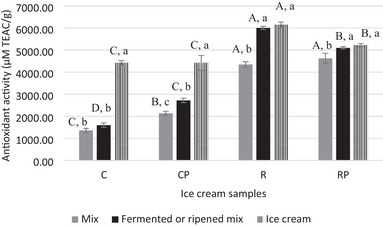
Changes in antioxidant activity of ice cream samples in relation to production parameters. Data are expressed as mean ± standard deviation (*n* = 2). C: with dairy milk and without *Bacillus coagulans*; CP: with dairy milk and with *B. coagulans*; R: with red kidney bean milk and without *B. coagulans*; RP: with red kidney bean milk and with *B. coagulans*. The CP and RP mixes were fermented, whereas the C and R mixes were ripened. Different uppercase letters signify significant differences across samples, whereas different lowercase letters denote differences across treatments (*p* ≤ 0.05).

The findings related to the antioxidant values of the samples produced from different milk sources are presented in Figure 3. IC samples made with RKBM exhibited significantly higher antioxidant activity (4349–4626 µM TEAC/g) compared to those made with dairy milk (*p* ≤ 0.05). This aligns with the observation that the same samples also exhibited the highest TPC values (Figure 2). The TPC of RKB has been reported to be responsible for its antioxidant activity (Zhu et al., 2024). Furthermore, RKBs are rich in lipophilic natural antioxidants such as tocopherols (Kan et al., 2017).

An increase in antioxidant activity was observed in mixes from both milk sources after the fermentation of probiotic mixes and the ripening of non‐probiotic mixes (*p* ≤ 0.05). These increases were more pronounced in mixes produced with RKBM. This is consistent with the increase in TPC observed following these processes. The rise in antioxidant activity after fermentation can also be attributed to the antioxidant compounds produced due to the metabolic activities of the probiotic *B. coagulans* ATCC 7050. Various studies have reported that exopolysaccharides produced by *B. coagulans* (Asianezhad et al., 2023), peptides formed from proteins by its proteolytic enzymes (Cao et al., 2020), and enzymes, such as superoxide dismutase and catalase, show antioxidant properties.

After the freezing process, an increase in antioxidant activity was observed in dairy ICs (*p* ≤ 0.05), whereas the antioxidant activity remained stable in vegan ICs (*p* > 0.05). In fact, the increase in TPC content in dairy IC samples with the freezing process was also sharp (Figure 2). On the other hand, normal and probiotic vegan ICs still demonstrated superiority over dairy ICs, with antioxidant activities of 6148 and 5225 µM TEAC/g, respectively (Figure 3). Likewise, Perera and Perera (2021) found that the use of coconut‐derived plant‐based milk in IC improved antioxidant activity. The findings not only emphasize the significance of producing plant‐based IC through fermentation but also demonstrate that RKBM is a promising alternative milk source for functional vegan IC production.

### 3.4 Probiotic viability and assessing the effects of production parameters on probiotic survival

**FIGURE 4:**
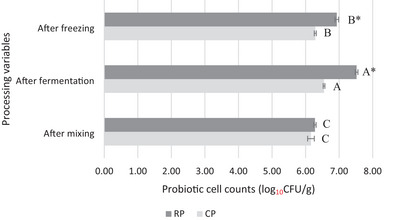
Probiotic cell counts in ice cream samples and their viability based on production parameters. Data are expressed as mean ± standard deviation (*n* = 2). CP: with dairy milk and with *Bacillus coagulans*; RP: with red kidney bean milk and with *B. coagulans*. Different uppercase letters signify significant differences across treatments (*p* ≤ 0.05). Differences between ice cream samples for each treatment were evaluated with the two‐sample independent *t*‐test, and stars indicate significant differences (*p* ≤ 0.05).

In this study, the viable counts of *B. coagulans* ATCC 7050 were determined following mixing, fermentation, and freezing processes. The findings regarding the probiotic viability of the samples are presented in Figure 4. After inoculation, it was determined that the CP and RP mix samples contained 6.16 and 6.28 log_10_CFU/g probiotic cells, respectively, with no significant difference (*p* > 0.05). Following fermentation, vegan ICs with RKBM showed the highest probiotic viability (7.52 log_10_CFU/g) (*p* ≤ 0.05), indicating RKBM as a suitable substrate for *B. coagulans* growth. RKBs are rich in carbohydrates, resistant starches, proteins, dietary fiber, and prebiotic oligosaccharides like raffinose, stachyose, and verbascose, which support probiotic growth (Kan et al., 2017; Siva et al., 2020). RKB polysaccharides were found to support the growth of *Lactobacillus* (*L*.) *fermentum* and *L. plantarum* (Jayamanohar et al., 2019), but this is the first study to evaluate their effects on *B. coagulans*. Although soy milk provides an optimal environment for *B. coagulans* (Christian et al., 2021), soy‐related allergies have driven interest in alternative legume‐based milk sources (Cabanillas et al., 2018). The results from this study suggest that the prebiotic compounds present in RKBs have the potential to support the growth of beneficial microorganisms like *B. coagulans*. Consequently, RKB presents an alternative solution for probiotic‐enriched food production while also offering the potential to reduce allergy risks.

During the IC processing, a reduction in the probiotic cell count was observed in both IC samples (*p* ≤ 0.05), with a probiotic viability of 6.29 log_10_CFU/g for dairy IC and 6.93 log_10_CFU/g for IC containing RKBM. Although *B. coagulans* is known to be resistant against adverse environmental conditions (Jafari et al., 2016), it has been reported that freezing induces stress on the probiotic *B. coagulans*, leading to an approximately 10% reduction in viable cell count. The decline in probiotic cell count after freezing can be attributed to the physical disruption of bacterial cells caused by ice crystals (Öztürk et al., 2018). In this study, the reduction in viable *B. coagulans* cell count within the IC matrix ranged from 3.97% to 7.85%. Akalın et al. (2024) determined the probiotic *Lactobacillus acidophilus* counts in almond milk, lupine milk, and hazelnut milk ICs as 5.41, 6.69, and 6.19 log_10_CFU/g, respectively. In another study, Kemsawasd and Chaikham (2020) recorded that probiotic viability in ICs made from riceberry and sesame‐riceberry milk was 4.30–4.96 log_10_CFU/g *for* *Lactobacillus* *casei* 01 and 5.43–6.25 log_10_CFU/g for *L. acidophilus* LA5. In the vegan ICs developed in this study, higher probiotic viability was achieved compared to the probiotic levels reported in the aforementioned studies. In addition to the supportive IC matrix for probiotic growth, the use of a spore‐forming bacterium (Payne, Bellmer, Jadeja et al., 2024) in our study may have provided a significant advantage in improving probiotic viability. It has been stated that to meet the minimum therapeutic level, probiotic microorganisms in a product must reach at least 6 log_10_CFU/g or mL (Terpou et al., 2019). In this study, all probiotic IC samples showed probiotic viability above 6 log_10_CFU/g. The ability of RKBM‐based matrices to maintain probiotic viability above the minimum therapeutic level suggests that they could be suitable not only for functional frozen desserts but also for a variety of probiotic‐enriched products in the food industry.

### 3.5 Sensory characteristics of vegan ICs compared to dairy‐based ICs

**FIGURE 5:**
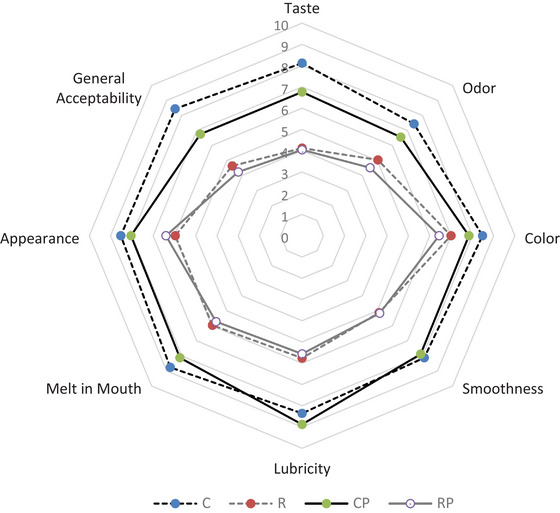
Sensory profile assessment of ice cream samples. Evaluations were conducted using a nine‐point hedonic scale (1 = extreme dislike, 9 = extreme like). C: with dairy milk and without *Bacillus coagulans*; CP: with dairy milk and with *B. coagulans*; R: with red kidney bean milk and without *B. coagulans*; RP: with red kidney bean milk and with *B. coagulans*.

The sensory characteristics of the ICs are shown in Figure 5. Dairy‐based IC received the highest taste score (8.12), whereas vegan ICs (R and RP) were rated lower (4.12 and 4.04, respectively). Studies have shown that plant‐based ICs, especially those made from legume‐based milks, often have less acceptable taste profiles due to consumer preferences (Akalın et al., 2024; Ghaderi et al., 2021). The earthy aroma of ICs made with RKBM may stem from aromatic compounds such as 1‐octen‐3‐ol, trimethylpyrazine, thymol, and 2‐ethyl‐3‐methylpyrazine, which are common in RKBs (Mishra et al., 2017) and likely contributed to lower taste scores. Probiotic addition did not significantly affect the taste of RKBM ICs. Dairy‐based ICs also scored highest for odor (7.44 and 6.56). However, probiotic addition reduced odor scores, possibly due to changes in the volatile profile, as noted in previous studies (Goktas et al., 2022). This observation aligns with findings reported by Payne et al. (2024), where the incorporation of probiotic *Bacillus subtilis* strains led to significant changes in sensory properties, including flavor and odor. The study highlighted that the production of organic acids, peptides, and other metabolic byproducts by probiotic cultures can alter the volatile profile and contribute to off‐flavors, such as bitterness or sourness.

The highest color scores were observed in the dairy ICs due to their higher brightness values (Table 4). In contrast, the slight red–brown hue from RKBM in vegan ICs (Table 4) led to lower color and appearance scores, as this color deviates from traditional IC hues. Additionally, IC samples containing RKBM showed lower smoothness and lubricity scores, likely due to the sandy texture often associated with plant‐based milk ICs (Akalın et al., 2024; Gorman et al., 2023). This texture negatively impacts smoothness, lubricity, and overall mouthfeel, as consumers prefer a creamy texture (Gorman et al., 2023). The dairy IC samples also achieved higher melt‐in‐mouth scores, consistent with reports that plant‐based milk ICs melt more slowly, leading to lower sensory scores (Akalın et al., 2024).

Dairy IC samples received the highest overall acceptability, with non‐probiotic dairy IC being the panelists’ favorite. Previous studies also show plain dairy or vanilla ICs are most preferred by consumers (Bekiroğlu & Özdemir, 2020). The lower sensory scores of RKBM ICs suggest consumers still favor dairy products. Although probiotics did not negatively affect RKBM ICs, they reduced taste, aroma, and acceptability in dairy versions. Therefore, plant‐based milk may be a better option for probiotic IC. Sensory enhancements for RKBM ICs could be achieved by incorporating natural flavor enhancers, which could significantly increase their consumer acceptance and broaden their potential applications in the food industry. Additionally, advanced sensory methodologies (ESM), such as Check‐All‐That‐Apply and Just‐About‐Right scaling, offer valuable tools for examining consumer perception (Ribeiro et al., 2024) and could play a pivotal role in future studies focusing on plant‐based formulations. By providing detailed insights into the sensory determinants of consumer preference, these methods can guide the optimization of flavor, texture, and overall sensory appeal in plant‐based ICs, ultimately enhancing their market acceptance and positioning.

## 4 CONCLUSION

The growing demand for plant‐based IC alternatives calls for innovative formulations. This study developed novel probiotic and non‐probiotic vegan ICs using RKBM. Functional, physicochemical, and sensory properties of these vegan ICs were compared to cow's milk ICs. RKBM showed higher probiotic viability after fermentation by supporting *B. coagulans* growth. Both cow's milk and RKBM ICs had similar titratable acidity after fermentation. RKBM ICs had lower brightness, higher red hue, and more yellow color. Vegan ICs also showed higher overrun, indicating improved technological properties. Fermentation, ripening, and freezing increased TPC in vegan ICs. Moreover, ripening or fermentation increased antioxidant activity in vegan ICs, which had higher radical scavenging activity than dairy ones. However, vegan ICs scored lower in taste, odor, color, and overall acceptability. Non‐probiotic dairy IC was the most preferred in terms of sensory test. Despite functional advantages of RKBM, more research is needed to enhance its sensory qualities in IC production, paving the way for its application in the food industry.

## AUTHOR CONTRIBUTIONS

**Emine Beyda Acar**: Formal analysis; investigation; methodology; writing—review and editing. **Talha Karahan**: Investigation; formal analysis; methodology; visualization. **Dora Mutlu**: Investigation; software; visualization; writing—review and editing. **Osman Sağdıç**: Writing—review and editing; visualization. **Hale İnci Öztürk**: Conceptualization; supervision; data curation; investigation; methodology; writing—original draft; writing—review and editing; visualization.

## CONFLICT OF INTEREST STATEMENT

The authors confirm that there are no identified conflicts of interest related to this publication.

## Data Availability

The data produced in this study are accessible through the corresponding author upon a request.
